# Implementing a Clinician-Built High-Dose Antipsychotic Therapy (HDAT) Calculator and Tracker: A Quality Improvement Project to Optimise HDAT Monitoring and Safe Prescribing

**DOI:** 10.1192/bjo.2025.10403

**Published:** 2025-06-20

**Authors:** Nurul Ain Mohd Nizam, Isabelle Tighe, Claire Butler, Paul Harris

**Affiliations:** Sussex Partnership NHS Foundation Trust, Brighton, United Kingdom

## Abstract

**Aims:** High-dose antipsychotic therapy (HDAT) carries an increased risk of adverse effects, including metabolic syndrome and extrapyramidal side effects, requiring regular monitoring. However, adherence remains inconsistent due to challenges in patient attendance, staff awareness, and varying monitoring intervals. This quality improvement project aimed to determine the prevalence of HDAT use in an assertive outreach team, assess adherence to the local trust HDAT monitoring guidelines, and implement a clinician-built HDAT Calculator and Tracker to improve monitoring efficiency.

**Methods:** In the absence of an electronic prescribing system, monitoring was routinely done manually. Therefore, clinicians created an HDAT Calculator and Tracker using Microsoft Excel, based on local trust HDAT monitoring guidelines and the Prescribing Observatory for Mental Health (POMH) Ready Reckoner Version 11, to automatically calculate and identify patients on HDAT, flag upcoming and overdue assessments, and facilitate monitoring. Data collected included the dates and results of the most recent electrocardiogram (ECG), blood tests, quantitative antipsychotic side effect assessments, and weight.

**Results:** Of 105 patients reviewed, 11 (10%) were identified as receiving HDAT at the time of data collection. 5 of the 11 patients on HDAT were in an inpatient setting. ECG and blood test compliance were both 91%, with reasons for missing parameters documented in all but one instance. 2 of 11 patients were due for their annual weight assessment. Notably, gaps were identified in the documentation of quantitative antipsychotic side effect assessments, with 3 of 11 patients lacking a recorded assessment and 4 of 8 overdue for their annual review.

Clinicians identified a significant challenge in monitoring patients after HDAT initiation due to varying intervals between required assessments (e.g. 3–4 days, 1 month and 3 months post HDAT initiation) and the complexity of ensuring timely follow-up. The HDAT Calculator and Tracker offered a systematic, sustainable solution, enabling clinicians to recognise upcoming assessments and plan timely interventions. Overall, feedback highlighted reduced administrative workload and increased confidence in ensuring continuity of care and safe prescribing.

**Conclusion:** This project highlights the importance of structured, ongoing monitoring in psychiatric practice and presents a model for improving safe prescribing in high-risk populations. Future steps include iterative updates to the tool as new knowledge emerges, increasing HDAT monitoring awareness within the multi-disciplinary team (particularly around the adverse effects of HDAT), joining up care with local physical health clinics, embedding the tool into routine clinical practice and integrating it with electronic patient records and prescribing systems currently under development.

